# When Nail Removal Is Not Benign: Iatrogenic Fractures Associated With Titanium Intramedullary Nail Removal

**DOI:** 10.7759/cureus.103009

**Published:** 2026-02-05

**Authors:** Sunil D Magadum, Mahesh Karthik, Ram Prasad Jasti, Kamal Asbar, Umesh Kumar Singh

**Affiliations:** 1 Orthopaedics, MIOT International Hospital, Chennai, IND

**Keywords:** iatrogenic fracture, implant osseointegration, risk factor for nail extraction, tips for safe nail removal, titanium nail removal

## Abstract

Intramedullary (IM) nailing is a standard treatment for diaphyseal fractures of long bones. Several factors influence bony ingrowth and osseointegration between the nail and bone. Removal of these nails may be required, especially in younger patients, for multiple indications. Bony ingrowth and osseointegration can cause resistance and complicate nail removal.

We report a series of three patients who had bony ingrowth and osseointegration within the nail and proximal metaphyseal bone, which complicated routine nail removal. One patient sustained an intertrochanteric femur fracture during the extraction of the femoral nail, while the other two patients sustained proximal tibial fractures during the extraction of the tibial nail.

All fractures that occurred during nail extraction healed over a period of three to four months without complications. In this case report, we have addressed the possible reasons for this complication and methods to prevent it. Removal of an IM nail requires adequate planning and instrumentation for safe extraction. Bony ingrowth and osseointegration can complicate routine nail removal. Surgeons should be mindful of these complications and be aware of different techniques for bone removal to ensure safe nail extraction.

## Introduction

Diaphyseal fractures of the femur and tibia are common fractures of long bones. Intramedullary (IM) nailing is the standard method of treatment for these diaphyseal fractures due to its advantages, which include a minimally invasive technique, shorter operative time, relatively stable fracture fixation, and early patient mobilisation [1,2]. However, factors such as nail design, the presence of flutes or grooves, the number and location of locking screws, the distance from the fracture site, and the material of the nail can influence the nail's ability to resist various forces [3,4]. Nail designs for diaphyseal fractures continue to evolve, primarily aiming to increase the stability of the construct. Recently, the use of titanium nails, nails with multiple locking options, and nails with variable diameters has become available in the market. Although the treatment of diaphyseal fractures with IM nailing is standardised, the protocol and timing for IM nail removal are not. In addition, the biocompatibility and surface characteristics of titanium nails can promote osseointegration, which may complicate implant removal. Hence, routine nail removal is not advised in asymptomatic patients; however, if indicated, it should be done in patients with a postoperative duration of more than 18 months, with evidence of robust bridging bone around the fracture and complete obliteration of the fracture line [5]. Relative indications for nail removal include pain, implant prominence, and soft tissue irritation. True medical indications include infection and intra-articular material [6,7]. Only a limited number of reports have described iatrogenic fractures during IM nail removal [8]. Im and Lee reported three refractures and two failures of nail removal among 30 patients undergoing extraction of ACE tibial nails, with younger age being the only factor significantly associated with difficulty in nail removal [9]. Takakuwa et al. described four cases of posterior tibial cortex fractures during removal of ACE tibial nails [10]. Despite IM nailing being a routine procedure, such complications during nail removal remain underreported and can have serious clinical consequences. We describe three such cases of iatrogenic fractures, highlighting similar complications that occurred during nail removal, and discuss the possible causes, risk factors, and methods to prevent this complication.

## Case presentation

Case one

**Figure 1:**
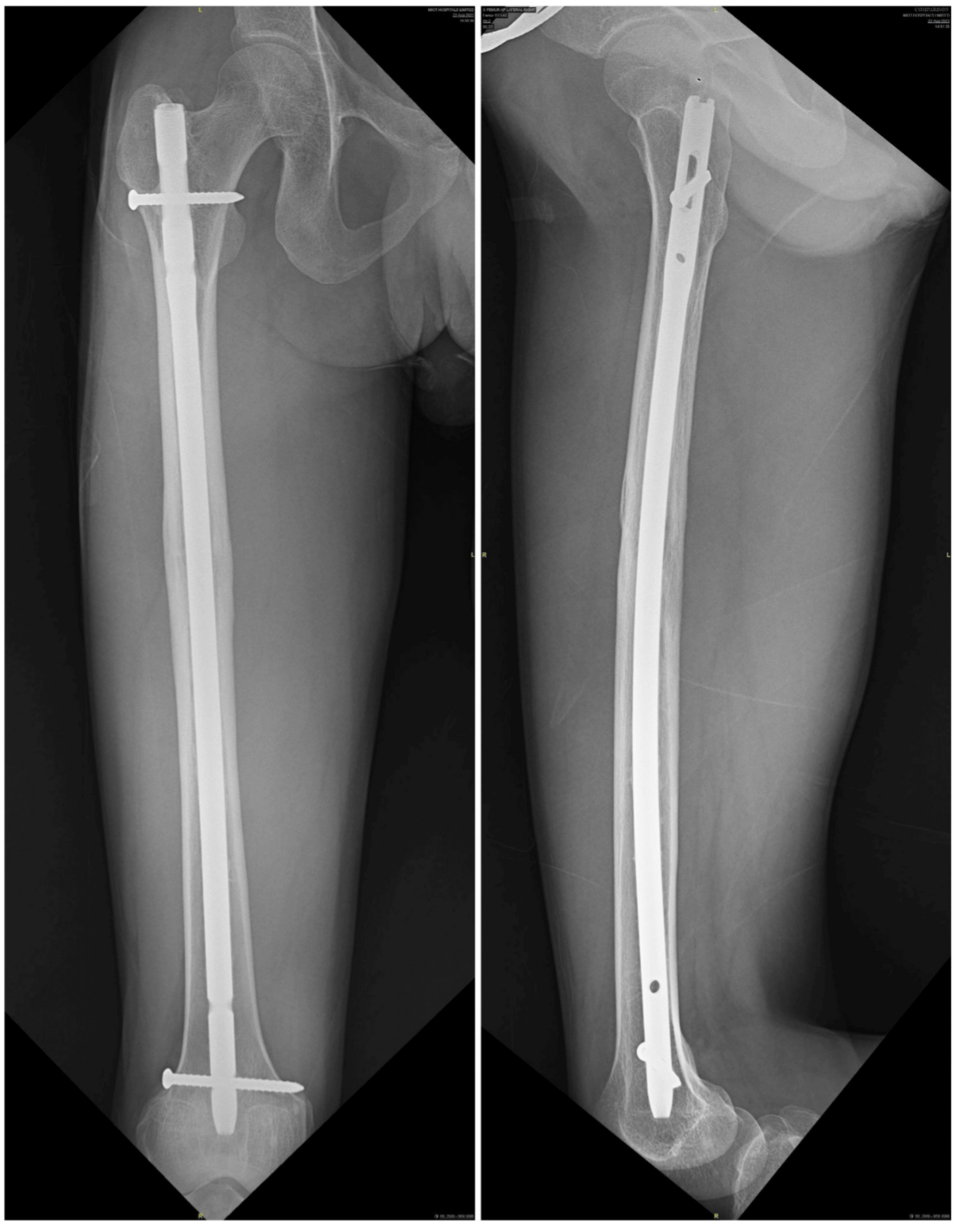
X-ray of the right femur showing healed shaft of the femur fracture, 11 years following intramedullary titanium femoral nail (10 mm in size) with one proximal locking screw in dynamic mode and two distal locking screws

**Figure 2:**
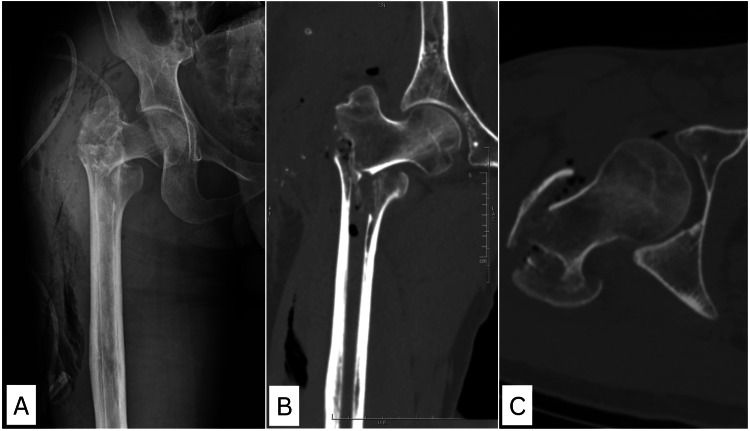
Immediate post-implant removal X-ray image (A) and CT images (B, C) of the right hip showing unstable comminuted trochanteric fracture of the femur

**Figure 3:**
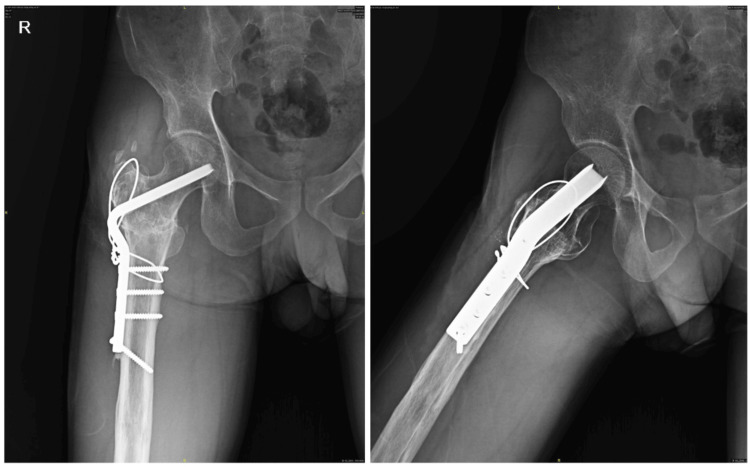
Four month follow-up X-ray of the right hip showing healed fracture after internal fixation with 120° angle blade plate

A 29-year-old gentleman underwent closed IM nailing of the right femur for a femoral shaft fracture 11 years ago. He came for a follow-up and requested implant removal. His X-ray showed that the fracture had united well (Figure 1). The nail used was a titanium femoral nail (10 mm in size) made of Ti-6Al-7Nb (TAN) with one proximal locking screw in the dynamic mode and one distal locking screw. Intraoperatively, routine steps of nail extraction were followed; however, nail extraction was difficult, and after repeated attempts at strong reverse hammering, the nail was removed. Unfortunately, his postoperative X-ray (Figure 2A) showed an unstable comminuted intertrochanteric fracture. CT was performed (Figures 2B, 2C) to evaluate the complete fracture geometry. After discussion with the patient and relatives, he underwent internal fixation with a 120° angle blade plate. No intraoperative neurovascular complications occurred. In view of local comminution at the nail entry point with trochanteric avulsion, surface fixation was preferred over IM fixation. The patient was mobilised with partial weight-bearing using two crutches, and after 12 weeks, full weight-bearing was allowed. Subsequently, the fracture healed in four months with no complications (Figure 3).

Case two

**Figure 4:**
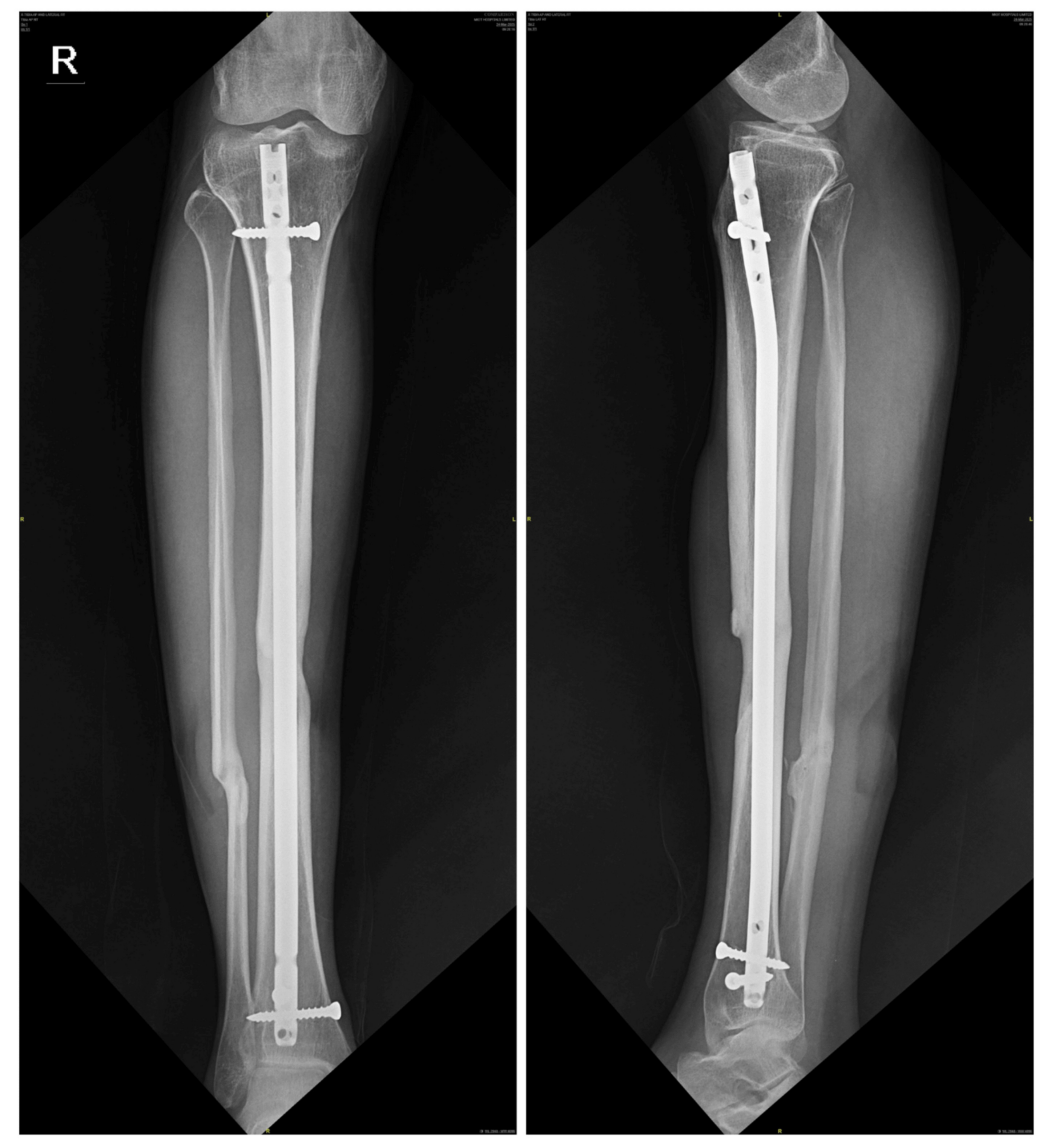
X-ray of the right tibia showing healed shaft of the tibia fracture, five years following cannulated tibial nail (11 mm in size) with one proximal locking screw in dynamic mode and two distal locking screws

**Figure 5:**
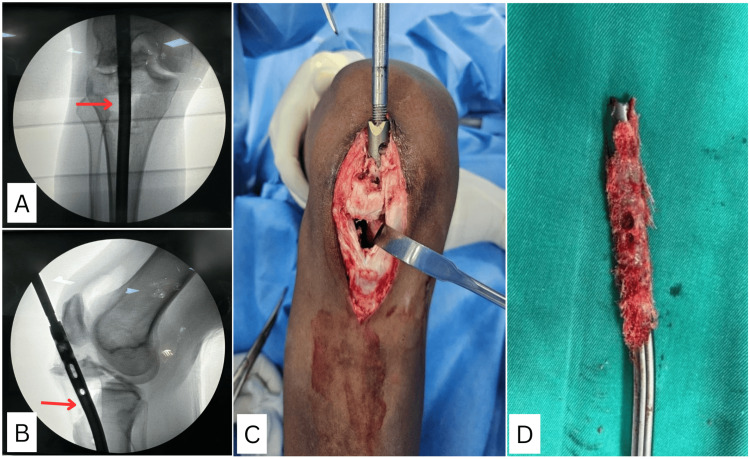
(A, B) Intraoperative C-arm images showing a proximal tibia fracture in the anterior cortex along with a tibial tuberosity fracture (red arrow). (C) Intraoperative clinical picture showing metaphyseal bone fragment adherent to the proximal portion of the nail. (D) Bony ingrowth seen along the four unused locking holes in the proximal part of the nail

**Figure 6:**
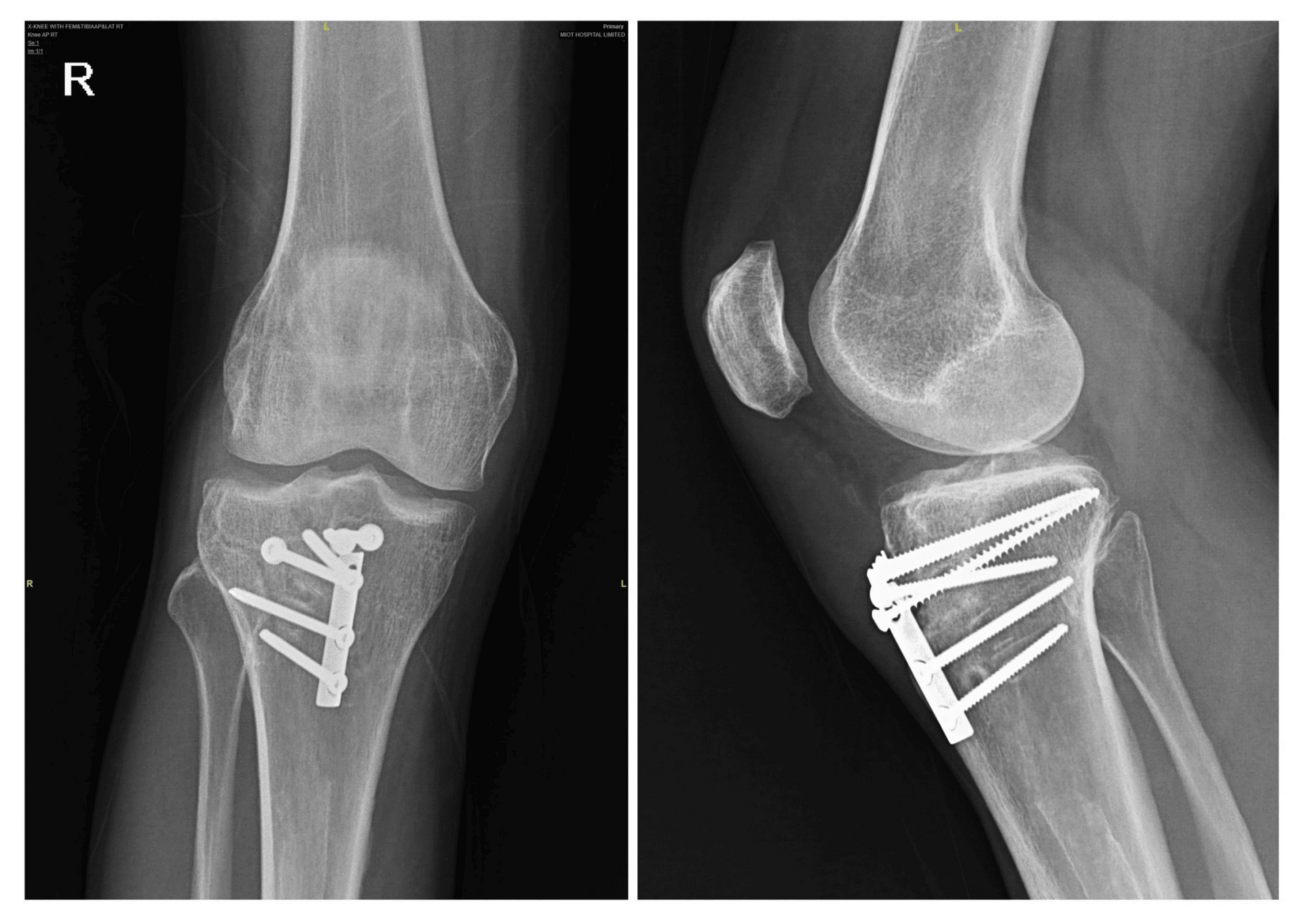
Four months' follow-up X-ray of the right knee showing a healed fracture after internal fixation

A 29-year-old gentleman underwent wound debridement and closed IM nailing for a compound Gustilo-Anderson grade IIIB fracture of both bones of the right leg five years ago. The nail used was an Expert tibial nail (11 mm × 345 mm in size) made of TAN with one proximal locking screw in the dynamic mode and two distal locking screws. The fracture healed in four months (Figure 4). He was admitted for implant removal after fracture union. While extracting the nail, deformity was noted in the anterior aspect of the knee, and a large chunk of metaphyseal bone came out along with the nail and was adherent to the nail near the proximal screw holes (Figures 5C, 5D). C-arm images showed a proximal tibia fracture on the anterior surface involving the anterior one-third of the articular surface along with the tibial tuberosity (Figures 5A, 5B). Hence, he underwent open reduction and internal fixation with a semitubular plate and interfragmentary screw fixation. No intraoperative neurovascular complications occurred. The patient was mobilised with partial weight-bearing using two crutches, and after 10 weeks, full weight-bearing was allowed. Subsequently, the fracture healed in four months with no complications and complete functional recovery (Figure 6).

Case three

**Figure 7:**
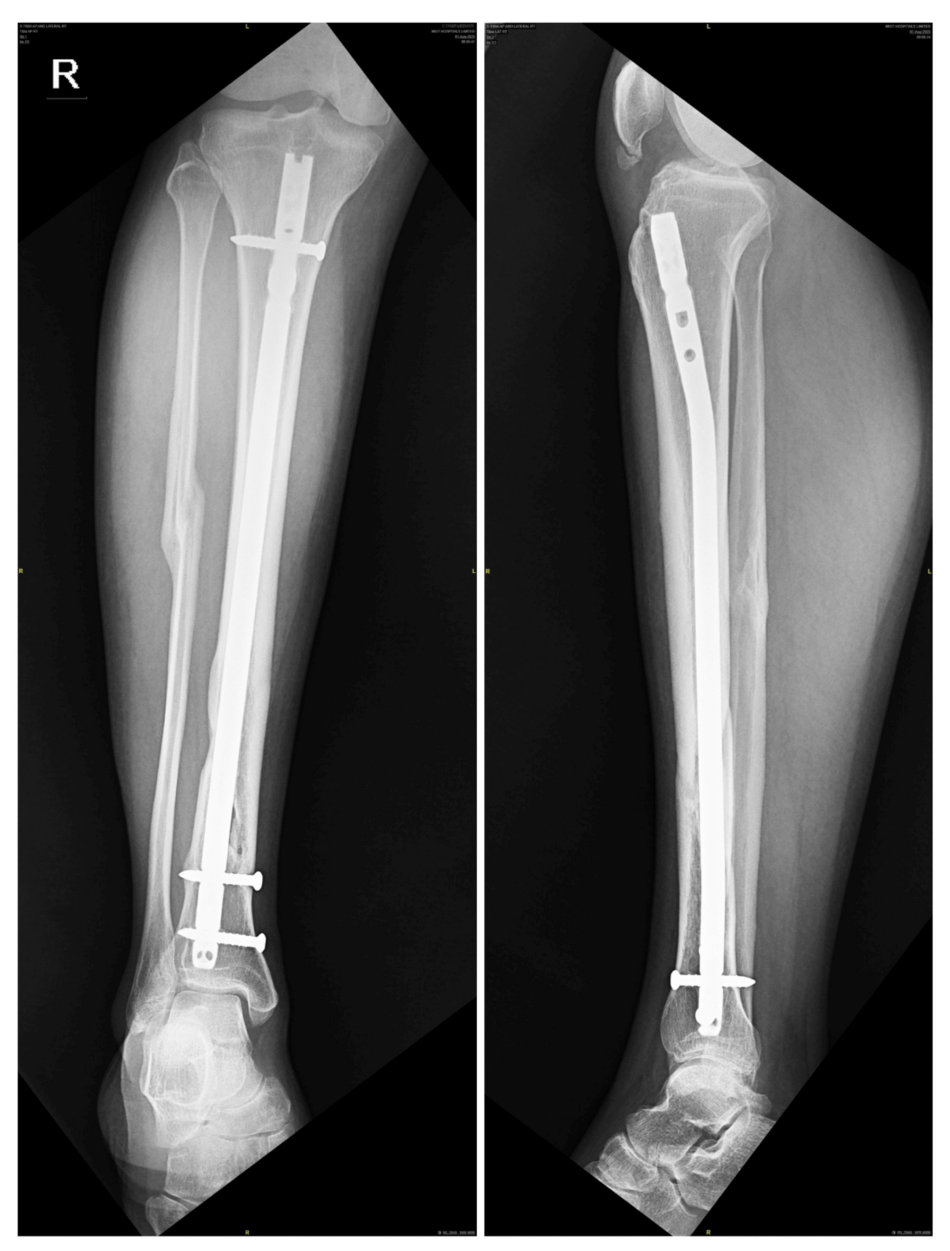
X-ray of the right tibia showing healed shaft of the tibia fracture, six years following cannulated tibial nail (10 mm in size) with one proximal locking screw in dynamic mode and two distal locking screws

**Figure 8:**
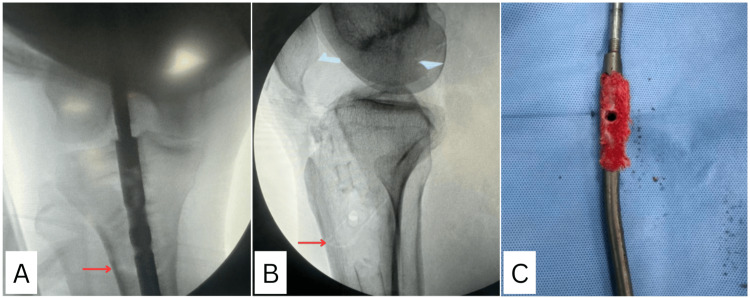
(A, B) Intraoperative C-arm images showing an undisplaced proximal tibia fracture (red arrow). (C) Bony ingrowth seen along the four unused locking holes in the proximal part of the nail

**Figure 9:**
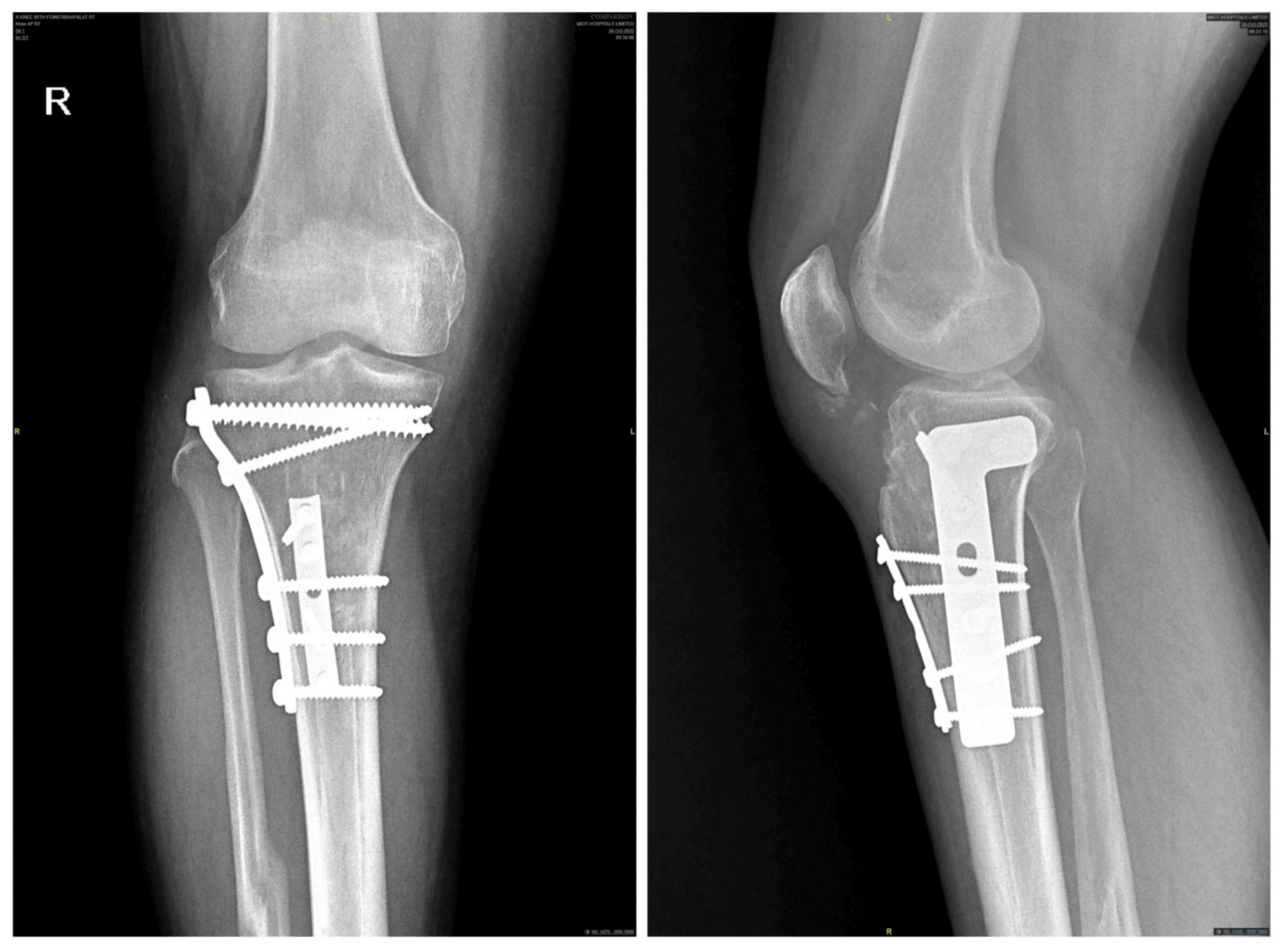
Three and half months' follow-up X-ray of the right knee showing healed fracture after internal fixation

A 30-year-old gentleman underwent IM nailing of the right tibia for a tibial shaft fracture six years ago. He came for a follow-up and requested nail removal. The X-ray showed that the fracture had healed, and he was planned for nail removal. The nail used was an Expert tibial nail (10 mm × 375 mm) made of TAN with one proximal locking screw in the dynamic mode and three distal locking screws (Figure 7). While extracting the nail, there was difficulty, and after repeated forceful hammering, the nail started moving; however, C-arm images showed an undisplaced oblique fracture extending from the anteroinferior cortex to the posterosuperior cortex (Figures 8A, 8B) at the level of the proximal locking screw site. He underwent internal fixation of the fracture with an L buttress plate and a one-third semitubular plate to allow early mobilisation. There were no intraoperative neurovascular complications. Similar to case two, we noticed a chunk of metaphyseal bone came along with the nail (Figure 8C). He was mobilised with partial weight-bearing ambulation for six weeks, followed by full weight-bearing walking. Subsequently, the fracture united by three and a half months with no complications (Figure 9).

A comparative summary of key variables across the three cases is presented in Table 1.

**Table 1:** Summary of key variables in the three cases

Variable	Case 1	Case 2	Case 3
Age (years)	29	29	30
Bone involved	Femur	Tibia	Tibia
Initial fracture type	Femoral shaft fracture	Compound IIIB both bones fracture	Tibial shaft fracture
Nail type	Titanium femoral nail	Expert tibial nail	Expert tibial nail
Nail material	Ti-6Al-7Nb (TAN)	Ti-6Al-7Nb (TAN)	Ti-6Al-7Nb (TAN)
Nail dimensions	10 x 400 mm	11 × 345 mm	10 × 375 mm
Number of proximal locking screws used	1 (dynamic)	1 (dynamic)	1 (dynamic)
Number of distal locking screws used	1	2	3
Number of unused proximal locking holes	1	4	4
Time from insertion to removal	11 years	5 years	6 years
Iatrogenic fracture	Intertrochanteric femur	Proximal tibia	Proximal tibia
Implant used	120° blade plate	Semitubular plate + screw	L buttress plate + 1/3 semitubular plate
Time to union	4 months	4 months	3 ½ months

## Discussion

Since 1939, surgical treatment with IM nails introduced by Kuntscher has been an effective and well-established method and has become the treatment of choice for diaphyseal fractures of long bones [11]. There has been an evolution in nail designs since then. The present generation of nails consists of titanium alloys with multiple locking options, variable diameters, and fluted designs. These features are added to increase the stability of the construct. The option of multiple locking extends the indications for nailing and increases rotational stability. However, this can also contribute to increased complications during nail removal. Usually, not all interlocking screw holes at the proximal end are used; therefore, bony ingrowth within these unused holes, which becomes consolidated and sclerotic over time, can engage the nail in the proximal metaphyseal bone and cause difficulty during nail removal. In our study, the femur had one proximal locking screw in the dynamic hole, thereby allowing bony ingrowth into the static and unused portion of the dynamic hole. In both cases of tibia, there were four unused locking holes in the proximal aspect. Bony ingrowth within these unused holes likely prevented nail extraction, and after strong and repeated reverse hammering, the nail, along with a metaphyseal cuff of bone, disengaged from the native bone, leading to an iatrogenic fracture. This bony ingrowth was noted in the proximal portion of the nail in all three cases.

Osseointegration refers to the incorporation of a nonbiological implant into host bone to establish direct and functional contact between the bone and the implant, thereby enabling physiologic load-bearing. Several factors can promote or hinder osseointegration. Factors such as composition and surface treatments, host bone quality, device suitability, and medications have been linked to both success and failure of osseointegration [12]. The biocompatibility of the implant material is also essential for effective osseointegration. Titanium has established itself as an ideal material for osseointegration due to its biocompatibility, corrosion resistance, and ability to generate an oxide adhesive layer on the outer surface, thus facilitating resistance to damage [13]. The surface film consists of TiO2 ( $\mathrm{Ti} + 2\mathrm{H}_2\mathrm{O} \rightarrow \mathrm{TiO}_2 + 4\mathrm{H}^+ + 4e^-$ )and is amorphous or of low crystallinity. The device finish also contributes to osseointegration. The microrough topography of common titanium alloys, such as TAN, which increases platelet and monocyte adhesion, favours osseointegration compared with smooth surfaces [14]. Implant osseointegration is important for clinical success in orthopaedic and dental applications, such as hip and dental implants; however, it is not essential in temporary fracture stabilisation and is not desired when implant removal is required. The use of titanium alloy in orthopaedic and trauma surgery has been advocated for its potential reduction in infection rates compared to stainless steel due to its improved biocompatibility and biomechanical properties. The surface of a medical implant is of great relevance since these implants are in direct contact with the host bone and fibrous tissue. The metaphyseal bone is cancellous and spongy, which allows osseointegration of implants with bone. This osseointegration produces significant resistance during implant extraction and can lead to complications such as fracture in the proximal metaphyseal region, which is evident by the cuff of cancellous bone wrapped around the proximal part of the nail after extraction. In all our cases, titanium TAN nails were used, and a large amount of osseointegration was observed in the proximal part of the nail, which was visible only after nail extraction (Figure 6).

Age is also an important factor influencing osseointegration. The younger the patient, the higher the chance of bony ingrowth, which can cause difficulty during nail removal. All patients in our case series were young [8]. 

Another potential problem during IM nail removal is related to nail cross-sectional design, which may consist of flutes and slots [4]. During the healing process, bony ingrowth through these flutes into the centre of the nail creates resistance to nail removal. Seligson stated that nail designs should ideally have a continuous cross-section [3]. If the nail design is uniform in cross-section, such resistance may not occur during nail extraction. All nails in our series had variable diameters and surface configurations.

The duration between primary surgery and nail extraction also plays an important role in the ease of nail removal. According to Boerger et al. [15], the mean time interval from radiological fracture union to nail removal was 16 months, with an average interval of 29 months from nail insertion to nail removal. In our study, femoral nail extraction was performed 11 years later, while the other two cases of tibial nail removal were performed after three and a half years and three years, respectively. Delayed implant removal may increase the risk of complications.

The possible ways to prevent the above complications include understanding the risk factors for difficult nail removal, such as younger patient age, duration since nail insertion more than 24 months, the presence of flutes or grooves on the nail surface, variable nail diameters, multiple unused proximal locking holes and material of the nail, and being pre-emptively prepared with the entire armamentarium, which includes multiple Kirschner wires of different diameters, drill bits of various sizes, hollow reamers, hollow mills of various diameters, bone gouge, periosteal elevators around the proximal part of the nail, etc. Additionally, nails and plates should be available on the table in case of an iatrogenic fracture. The patient should be counselled regarding the potential difficulties in nail removal, and appropriate informed consent should be obtained before surgery. Preoperative radiographs should be critically analysed to identify the implant design, number of unused holes, device finish, differential diameter, and metallurgy. When bony ingrowth and osseointegration are suspected, drilling and breaking the bony ingrowth within the proximal aspect of the nail, and unused holes using a long 3 mm Kirschner wire or 3.2 mm drill bit, helps ease nail extraction and avoid possible fracture. K-wires, bone gouges, periosteal elevators, and hollow reamers can be used to remove bone around the nail entry point and proximal part of the nail before definitive nail extraction and reverse hammering are attempted. Forward and backward hammering of the nail can also be attempted, rather than relying only on reverse hammering to break the osseointegration. The surgeon should refrain from excessive hammering, carefully analyse the reason for the difficult extraction, and attempt to address it using the above-mentioned methods. Given the descriptive nature of this case series, the findings cannot be subjected to statistical analysis nor can they be generalised in view of the limited number of patients.

## Conclusions

There is no clear consensus on the exact timing of nail extraction. Nail extraction may become challenging when the duration since nail insertion exceeds two years, particularly in young patients with titanium nails, a greater number of unused proximal locking holes, and variable nail diameters with flutes on the outer surface, due to bony ingrowth and osseointegration. Adequate preparation of the proximal part (nail entry point), both inside and outside the nail, is essential for safe nail removal to free the metaphyseal bone from the nail. Nail extraction should be planned early, as soon as remodelling is complete, which typically occurs 18-24 months after the procedure. Late extraction beyond 24 months in a young patient with a titanium implant should be done with proper precautions. Adequate planning and instrumentation must be ensured during surgery to address intraoperative challenges.
